# Introducing the UK Crop Microbiome Cryobank data resource, AgMicrobiomeBase, with case studies and methods on metabarcoding analyses

**DOI:** 10.1186/s40793-025-00768-5

**Published:** 2025-08-21

**Authors:** Payton To Yau, Rodrigo G. Taketani, J. Miguel Bonnin, Helen Stewart, Catriona M.A. Thompson, Ian M. Clark, Tim H. Mauchline, Jacob G. Malone, Matthew J. Ryan, Susan Jones, Nicola Holden

**Affiliations:** 1 Department of Rural Land Use, Scotland’s Rural College, Aberdeen, AB21 9YA UK; 2 Rothamsted Research, West Common, Harpenden, AL5 2JQ UK; 3 CAB International, Silwood Park, Ascot, SL5 7PY UK; 4 John Innes Centre, Norwich Research Park, Norwich, NR4 7UH UK; 5 Information and Computational Science Department, The James Hutton Institute, Dundee, DD2 5DA UK

**Keywords:** Microbiome, Cryobank, Crop, Soil, Rhizosphere, Metabarcoding

## Abstract

**Background:**

Here, we describe AgMicrobiomeBase as an output of the UK Crop Microbiome Cryobank (UKCMCB) project, including details of the underlying meta-barcode sequence-based methods and three microbiome analysis case studies. The UKCMCB links genomic datasets and associated soil metadata with a cryobank collection of samples, for six economically significant crops: fava bean (*Vicia faba)*, oil seed rape (*Brassica napus*), spring barley (*Hordeum vulgare*), spring oats (*Avena sativa)*, spring wheat (*Triticum aestivum*) and sugar beet (*Beta vulgaris*). The crops were grown in nine agricultural soils from the UK, representing three major soil texture classes. The UKCMCB is a scalable sequence-based data catalogue linked to a cryo-preserved sample collection.

**Results:**

The focus of this paper is the amplicon sequencing, associated bioinformatics workflows, and development of the project data catalogue. Short-read amplicon sequencing (16 S rRNA gene and ITS region) was implemented to describe the rhizosphere and bulk soil communities, for the multiple crop-soil combinations. Three case studies illustrate how different biological questions in phytobiome research can be addressed using this data resource. The three case studies illustrate how to (1) determine the impact of soil texture and location on microbiome composition, (2) determine a core microbiome for a single crop across different soil types, and (3) analyse a single genus, *Fusarium* within a single crop microbiome. The UKCMCB data catalogue AgMicroBiomeBase (https://agmicrobiomebase.org/data) links the sequence-based data with soil metadata and to cryopreserved samples.

**Conclusions:**

The UKCMCB provides baseline data and resources to enable researchers to assess the impact of soil type, location and crop type variables on crop soil microbiomes. The resource can be used to address biological questions and cross-study comparisons. Development of the UKCMCB will continue with the addition of metagenome and bacterial isolate genomic sequence data and has the potential to integrate additional data types including microbial phenotypes and synthetic microbial communities.

**Supplementary Information:**

The online version contains supplementary material available at 10.1186/s40793-025-00768-5.

## Background

Soil is an environment with one of the highest levels of microbe density and diversity. Microbiomes have been well described for a range of biological hosts and environmental habitats, transforming our understanding of biological functions and dysbiosis, and generating the concept of species or specific tissues as ecosystems and holobionts [1]. An understanding of microbial communities associated with plants enable discoveries about plant-microbe interactions [2] and plant health, and potentially have wide applications for increasing crop plant productivity [3]. To date, research emphasis has been on the microbiomes of major crop species like wheat, rice and maize [4, 5]. Less attention has been given to other economically significant crop species grown in the United Kingdom (UK). Additional crops have mainly been investigated in different contexts, such as nitrogen fixation for legumes [6] or tillage systems for barley [7]. Agricultural practices influence the soil microbiota, impacting crop health, yet baseline data can be difficult to attain and/or be very diverse [8]. In addition, there is a need to better understand the soil microbiota on a landscape and countrywide scale. Resources on crop plant microbiomes enable multiple types of research questions to be answered, and aid in development of sustainable agriculture practices.

Access to microbiome data is facilitated by nucleotide archives. In Europe, microbiome datasets are accessed from MGnify [9], which stores diverse data from different environmental hosts and habitats, including plants and soils. Alternative resources such as microbiome atlases also collate datasets for context-specific investigations or provide more generalised information [10, 11]. Such resources contribute significantly to answering sequence-based research questions. However, there is a need to link rich, contextual metadata to the sequence data, as well as to physical resources held in biobanks to maximise the utility of all data types.

The UK Crop Microbiome Cryobank project (UKCMCB) has established a cryopreserved resource of fully characterised material from soil and rhizosphere crop microbiomes [12]. Here, we describe AgMicrobiomeBase as a data resource output of the UKCMCB project, including details of the underlying meta-barcode sequence-based methods and illustrated by three microbiome analysis case studies. The resource output comprises material from six economically significant crops: fava bean (*Vicia faba)*, oil seed rape (*Brassica napus*), spring barley (*Hordeum vulgare*), spring oats (*Avena sativa)*, spring wheat (*Triticum aestivum*) and sugar beet (*Beta vulgaris*), grown in nine UK agricultural soils of different textural types. The data collected included DNA sequences, soil chemistry, physical soil parameters, field locations, agricultural history of the fields, crop species and crop genotypes. The rhizosphere microbiota was characterised using a combination of sequence-independent and culture-based approaches.

Multiple sequence-based data were obtained to link genomic datasets with cryo-preserved samples for each of the crop-soil combinations. Here, the amplicon sequence dataset is described alongside three case studies that illustrate how to (1) determine the impact of soil type and location on microbiome composition, (2) determine a core microbiome for a single crop across different soil types, and (3) analyse a single genus, (*Fusarium)* within a single crop microbiome. The overall aim is to demonstrate how the resource can be utilised to address specific research questions on crop microbiomes.

## Methods

### Soil sample collection

The soil sample collection and pot experiment have been described previously [12]. In brief, the pot experiment comprised six crops (Table 1) and nine agricultural soils (Table 2). The soils were selected to represent different textural types, from locations across the UK, provided by the BBSRC ASSIST programme network, and farms affiliated with Rothamsted Research, the James Hutton Institute and SRUC [12]. Each crop-soil combination, and no-crop bulk soil control comprised five biological replicates, generating a total of 270 crop-soil samples and 45 no-crop control samples. The 6 crops planted in the 9 agricultural soils gave 54 crop-soil combinations.

**Table 1:** Summary information for the six crops used for the large-scale pot experiment as part of the UKCMCB project

Common Name (Project Code)	Species name	Genotype
Fava Beans (FB)	*Vicia faba*	Linx
Oilseed Rape (OR)	*Brassica napus*	Campus
Spring Barley (SB)	*Hordeum vulgare*	RGT-Planet
Spring Oats (SO)	*Avena sativa*	WPB Elyann
Spring Wheat (SW)	*Triticum aestivum*	Mulika
Sugar Beet (SU)	*Beta vulgaris*	Dages

**Table 2:** Summary information for the agricultural soils collected for large-scale pot experiment as part of the UKCMCB project

Textural Class	UK County	Project Code	Latitude	Longitude
Clay loam	Borders	CL-BO	55.53806	-2.63665
Clay loam	Yorkshire	CL-YO	54.20593	-1.05670
Clay	Buckinghamshire	CY-BU	51.81865	-0.91014
Clay	Yorkshire	CY-YO	54.21591	-1.06591
Silty clay loam	Shropshire	SC-SH	52.49369	-2.47887
Sandy loam	Angus	SL-AN	56.48791	-3.13731
Sandy loam	Bedfordshire	SL-BE	52.00040	-0.61427
Sandy loam	Shropshire	SL-SH	52.42665	-2.47928
Silty clay loam	Hertfordshire	SC-HE	51.81805	-0.40552

### Amplicon library preparation & sequencing

The microbial composition of the rhizosphere and bulk soil were determined using amplicon sequencing from the V3-V4 region of the 16 S ribosomal RNA gene for bacteria and the variable intergenic ITS-1 region for fungi. 16 S rRNA gene amplicon sequencing was conducted on all six crops (Table 2) and ITS amplicon sequencing was conducted on spring wheat only. PCR controls included the no-template amplification control and a positive amplification control of a synthetic community, comprising purified gDNA from *E. coli* and presumptive plant-associated *Bacillus* spp. and *Pseudomonas* spp. isolates. The positive control ASV designations at the Genus level were *Escherichia-Shigella*,* Arthrobacter* and *Pseudomonas* respectively. Normalisation between sequencing runs was applied using a statistical technique to control for batch effects (see below).

DNA was isolated for amplicon library preparation from 250 mg aliquots of each soil sample using DNeasy PowerSoil Pro Kits (Qiagen, UK) according to the manufacturer’s instructions. The DNA was quantified using QuantiFluor^®^ ONE dsDNA System kits (Promega, UK) and normalised to ~ 5 ng/µL. The 16S rRNA gene sequences were amplified from the V3-V4 region using forward primer V3: 5’-CCTACGGGNGGCWGCAG-3’ and reverse primer V4: 5’- GACTACHVGGGTATCTAATCC-3’. The ITS rRNA spacer DNA sequences were amplified from the ITS1 region using forward primer ITS1-Fl2: 5’-GAACCWGCGGARGGATCA-3’ [13] and reverse primer ITS2: 5’-GCTGCGTTCTTCATCGATGC-3’ [14], both sets in 25 µl reaction volumes.

The PCR amplification was carried out in a 5PrimeG/02Techne Thermal cycler (Alpha Laboratories, UK) using KAPA HiFi HotStart ReadyMixPCR Kits (Roche Life Sciences, UK). and Illumina protocols [15]. The cycling conditions for both the 16 S rRNA gene and the ITS amplicon reactions were 95 °C for 3 min; 25 cycles consisting of 30 s at 95 °C, 30 s at 55 °C, and 30 s at 72 °C; with a final extension at 72 °C for 5 min. A Nextera Flex DNA Library kit (Illumina, UK) was used to generate the sequencing libraries, by addition of indices for 96-sample multiplexing, reaction clean up, normalisation, and pooling, as per the manufacturer’s instructions. Aliquots of 1 µl of the indexed PCR products were quantified using the QuantiFluor^®^ ONE dsDNA System kit (Promega, UK) and the final concentration measured on a GloMax explorer (Promega, UK). The libraries were then diluted with 10 mM of Tris buffer (pH 8.5) and validated using an Agilent 2100 Bioanalyzer (Agilent Technologies Ltd, UK). The libraries were sequenced on an Illumina MiSeq machine at the James Hutton Institute (Dundee, UK) using MiSeq reagent kit v2 (500 cycles) and read length 250 bp paired end.

### Amplicon sequencing data analysis

**Fig. 1:**
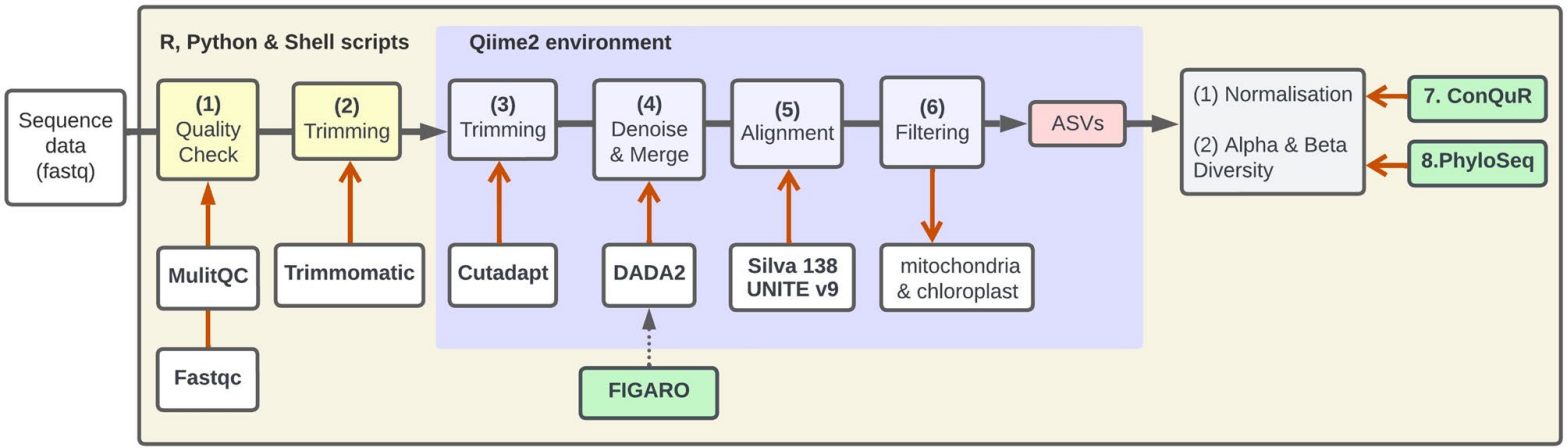
The amplicon sequencing analysis workflow. (**1**) FastQC [11] and MultiQC [6] were used for initial quality checking. (**2**) Trimmomatic [20] was used to remove low quality sequence start and end points. Within Qiime2 [8] (**3**) Cutadapt [12] was implemented for adapter trimming. (**4**) DADA2 for denoising and merging the reads, with parameters estimated using FIGARO [13, 14]. (**5**) Amplicon sequence variants were then defined and taxon assignments made using the Silva v138 [15] and UNITE v9 [26] reference databases. A final step within Qiime was the filtering of ASVs identified as mitochondrial and chloroplastic. (**7**) ConQuR [27] was then used for batch effect correction and the (**8**) Phyloseq R package [28] for the calculation of alpha and beta diversity

The workflow used for the amplicon sequence analysis is summarised in Fig. 1 and the code and the parameters used are available on GitHub [16]. In summary, the raw sequence reads were assessed using FastQC [17, 18] and the results combined across multiple samples using MultiQC [19]. Trimmomatic [20] was used to trim the overall length of the sequences to remove low quality start and end points as determined from FastQC. A Qiime2 (v2023.5) [21] workflow was used for further quality control, denoising, merging and taxonomic assignment. Within Qiime2 Cutadapt [22] was used to trim forward and reverse adaptor sequences. DADA2 [23] was used for filtering, dereplication, chimera identification and merging paired-end reads. DADA2 models and corrects Illumina based sequence errors, enabling a robust identification of biological variants. The filtering and merging parameters for DADA2 were determined using FIGARO [24], which models the error rate for each sequence to find optimal trimming sites that will maximize read retention. The complete Qiime2 workflow gave representative amplicon sequence variants for each sample. Reference databases Silva (v 138) and UNITE (v 9.0) [25, 26] were used for taxonomic assignment of the 16 S rRNA gene and ITS amplicons, respectively. Reads were classified by taxon using Qiime2’s feature classifier classify-sklearn and taxonomic filtering conducted to exclude mitochondria and chloroplasts sequences.

This project comprised a total of 315 sequenced samples distributed over four multiplexed sequencing runs using 96-well plates. ConQuR [27], that uses a two-part quantile regression model, was then used to control for potential batch effects.

After batch effect correction, variation within and between sample sets was assessed by calculating Shannon alpha and beta diversity based on the Bray-Curtis distance measure, respectively, using the Phyloseq package (v1.46.0) [28] in R.

### Data deposition and data catalogue

The raw sequence reads for each rhizosphere sample were uploaded to the European Nucleotide Archive (ENA) [29] using the genomic Standards Consortium (GSC) checklist GSC MIxS plant associated template (ERC000020). The bulk soil (no crop) control sample raw reads were uploaded using GSC MIxS soil (ERC000020) checklist template. The UKCMCB project comprises data for bulk soils and crop rhizospheres, which are linked in a parent (soil) - child (plant) relationship. This parent-child relationship has been established through submission of our own relationship template to BioSamples [30]. This template is available as part of our project catalogue AgMicrobiomeBase [31].

**Fig. 2:**
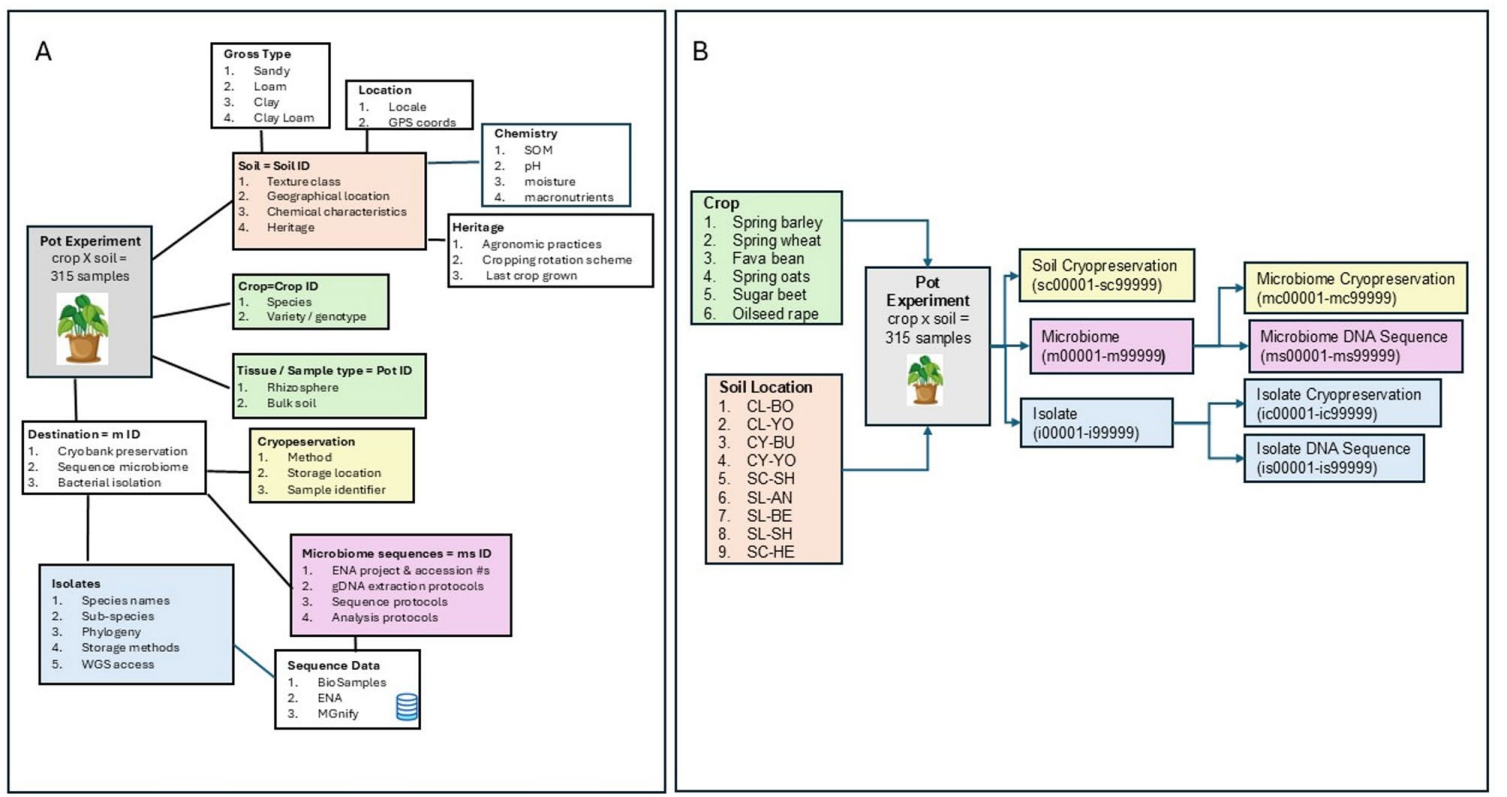
Diagram to summarise the data types and identifiers required to integrate data within the Agmicrobiomebase data catalogue. (**A**) Relationships between the multiple data types. The data types included those related to the soil, the crop and the cryopreservation(**B**) The unique identifiers assigned for use within the agmicrobiome catalogue and deposited with the sequence data into the public databases. The colours in each panel (A and B) link the data type and the identifiers

In addition to parent-child data relationships, the UKCMCB project has multiple data types. Here the 16 S rRNA gene and ITS data is described, but the project also includes soil metagenomic sequences, whole genome bacterial isolate sequences, phenotypic data, as well as chemical and physical soil properties (Fig. 2A). All project metadata was stored in Excel spreadsheets, with each project consortium partner contributing their metadata to one spreadsheet version. Each partner spreadsheet was then merged to create a complete project data catalogue. The key to the data merging was the use of unique identifiers for each data type (Fig. 2B). Queries on the data catalogue are visualised, and data reports created at agmicrobioembase.org using Microsoft PowerBI.

### Case studies

To demonstrate the utility of our data catalogue (which combines sample meta data with ENA sequence run identifiers (ERR)), we present three case studies that address different biological questions. The first examined the influence of soil type / location on a single crop species on bacterial taxonomies; the second determined the core microbiome for the 16 S rRNA gene amplicon dataset, for a single crop in all soil / location combinations; and the third assessed the fungal taxonomy for a sub-set of soil types / locations on a single crop species.

## Results

### Sequence data availability

The UKCMCB amplicon sequence data was deposited into the ENA under study accession PRJEB58189. A summary of the raw, trimmed and merged read counts for the complete dataset is shown in Table S1.

### The data catalogue

To enable data management and access to the UKCMCB project data in a way that met FAIR principles [32] a data catalogue, AgMicrobiomeBase [31], was created. It is a public website which links the genomic resources with extensive soil metadata [12]. Whilst the sequence data can be uploaded to public repositories, there was a need to create our own data catalogue to act as a hub for the whole project. Our catalogue creates a link between the sequence data and the cryopreserved samples: a unique feature of the project. Figure 2 outlines the relationships between the multiple data types produced by the UKCMCB project and the unique identifiers that link data within the data catalogue. The catalogue allows users to download the metadata for subsets of the amplicon and metagenome data as a spreadsheet. The metadata spreadsheet includes the ENA run accessions (ERR), allowing access to the raw sequence data with a knowledge of the sample structure (how samples relate to each other), something usually only obtained after reference to a publication.

### Batch effect correction

**Fig. 3:**
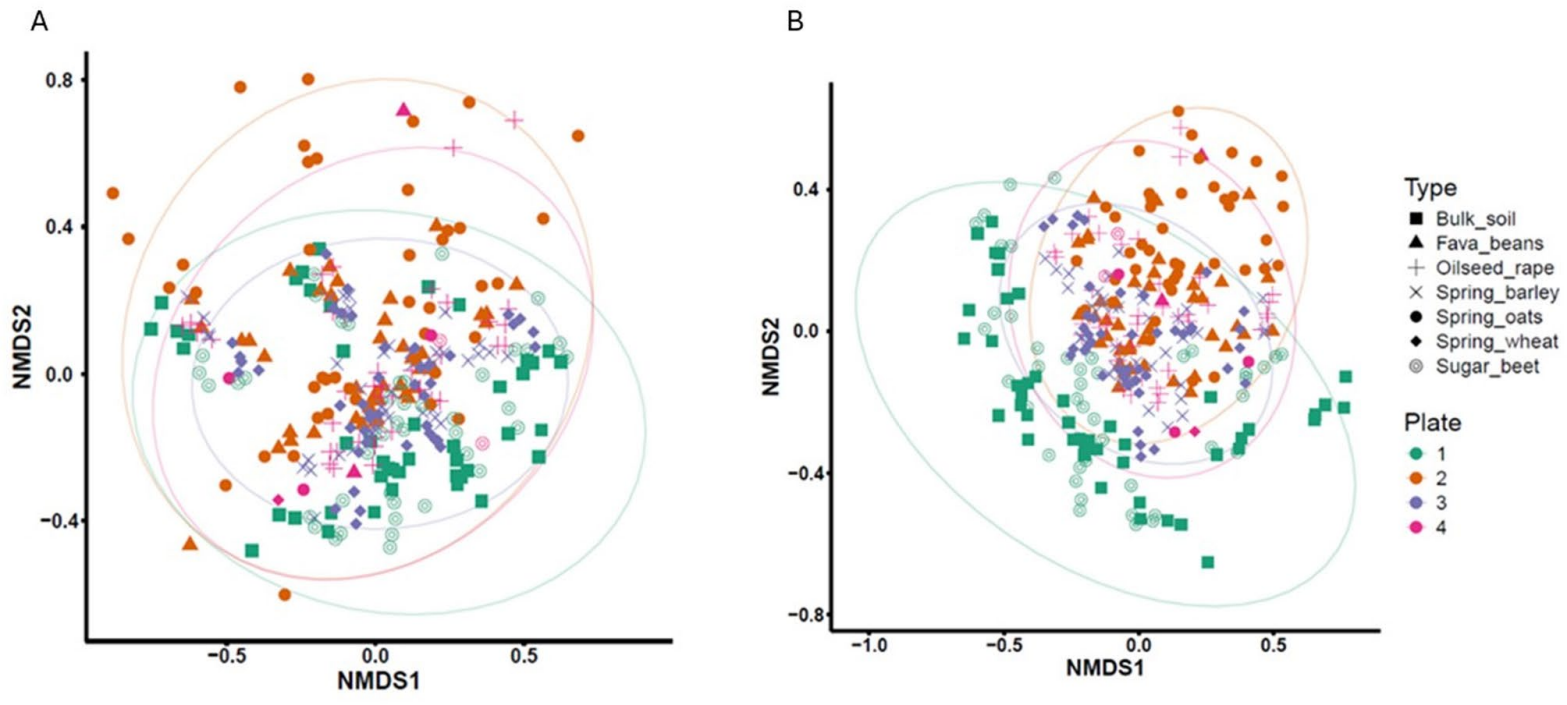
Taxonomic distribution of the 16 S rRNA gene ASVs displayed on an ordinance plot for β diversity based on the Bray-Curtis distance (**A**) before normalisation and (**B**) after normalisation using ConQuR. The datapoints are displayed by sequencing plate batch (1–4: colours) and by crop type (symbols)

To account for difference between multiple sequencing runs (*n* = 4), a batch correction was applied. The impact was evident for the taxonomic distributions before and after batch correction with ConQuR, as shown in a non-metric multidimensional scaling (NMDS) ordination plot (Fig. 3). The overall effect was a reduction in the variation between individual samples. Prior to normalisation there was some evidence for a batch effect (Fig. 3A), especially for plate 1 (green) and plate 2 (orange). Application of the ConQuR normalisation tightened the distributions for plates 2,3 and 4 with crop-dependent groupings more evident (Fig. 3B). After normalisation, there was still an apparent batch effect for Plate 1, which might be explained by the grouping and distribution of the bulk soil / no-crop control samples on this plate.

**Fig. 4:**
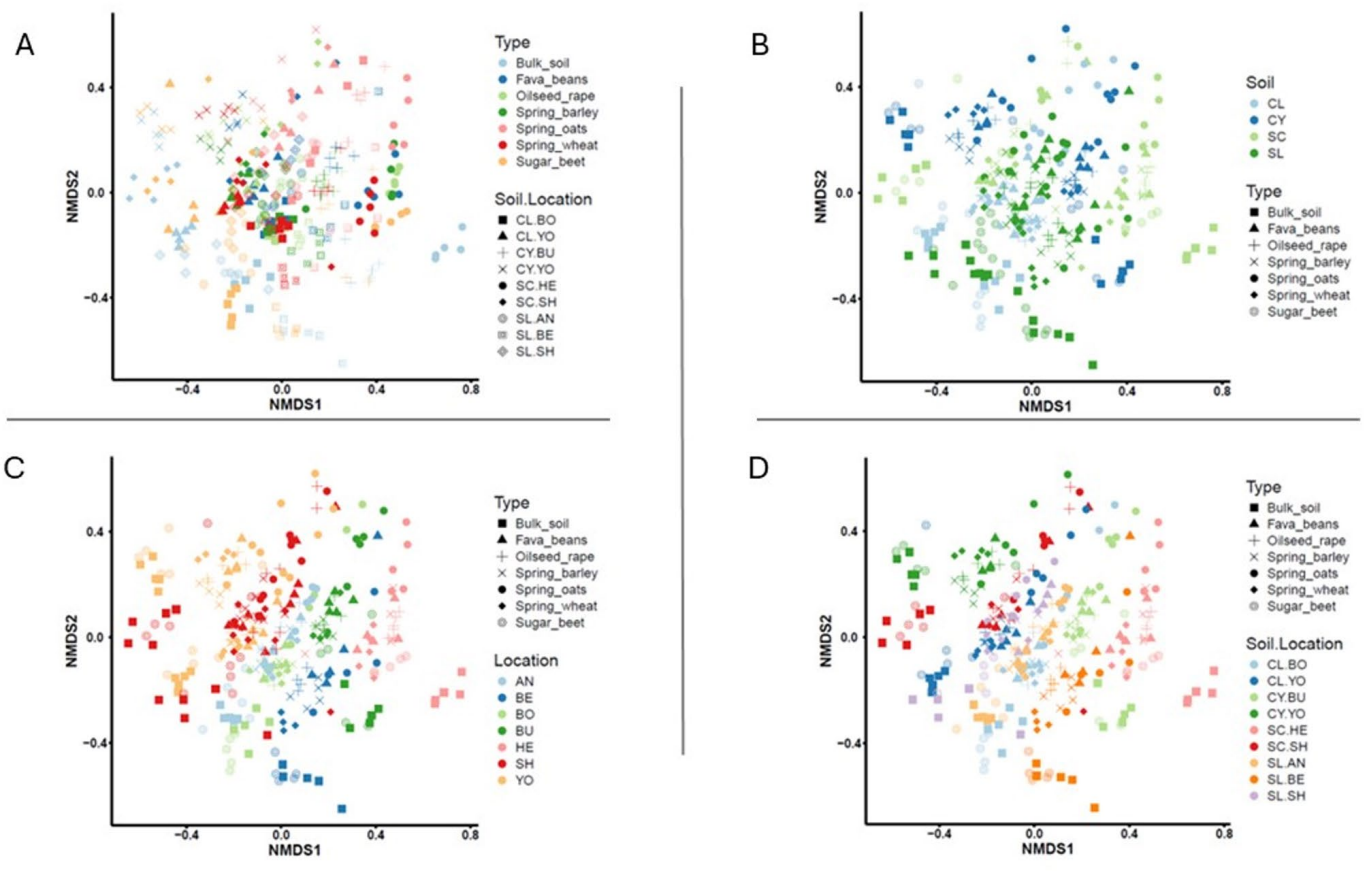
Location driven microbe recruitment. NMDS ordination plots of beta diversity based on the Bray-Curtis distance for the complete dataset (6 crops and 9 agricultural soils). Colours based on (**A**) crop (**B**) soil type (**C**) location (**D**) soil type/location. See Table 2 for soil legend

Assessment of the variation between sample sets showed distinct grouping by soil type / location combination for the different crops (Fig. 4D). Different visualisations highlight the influence for each variable of crop species (Fig. 4A), location (Fig. 4C) and soil textural class (Fig. 4B). Figure 4 also provides evidence for concurrence of sample replicates for each soil type / location - crop species combination.

### Case study one: assessing the impact of soil type and location on the bacterial microbiome composition in the sugar beet rhizosphere

**Fig. 5:**
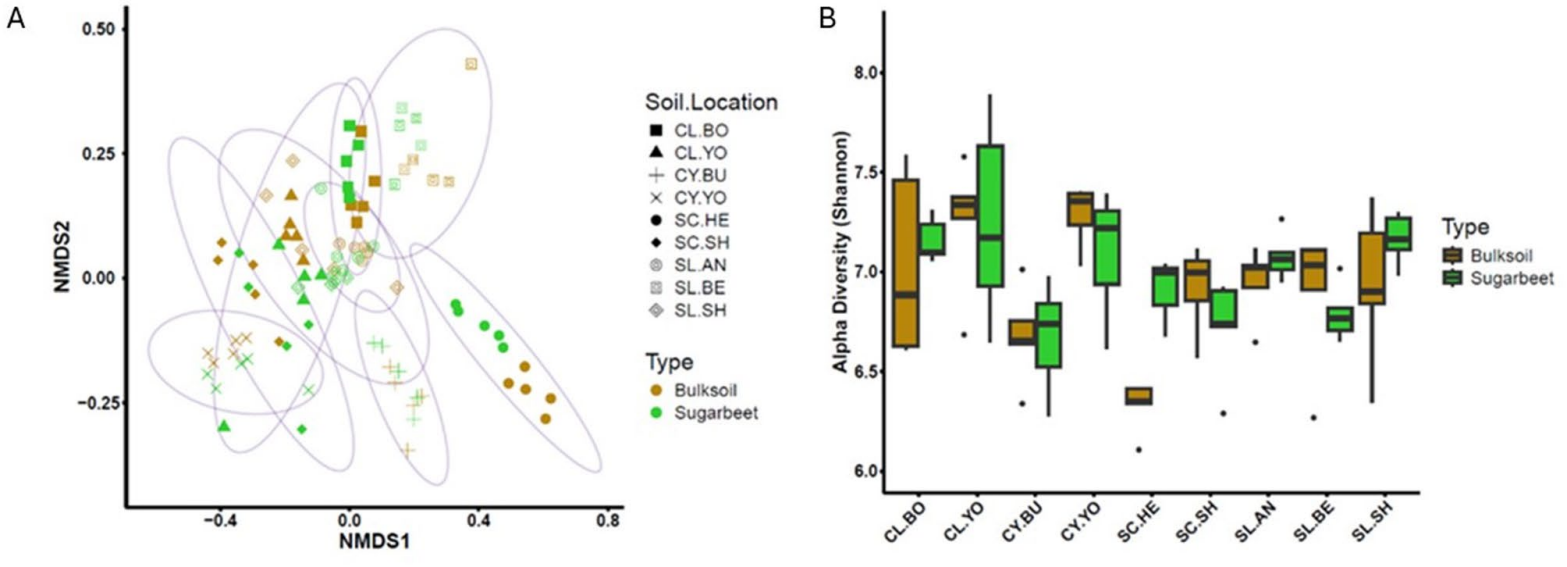
Case study 1: assessing the impact of soil type and location on the bacterial microbiome composition of the sugar beet rhizosphere. (**A**) NMDS ordination plot of beta diversity based on the Bray-Curtis distance for sugar beet rhizosphere (green) and bulk soil (brown) microbiomes of nine agricultural soils. The symbols and ellipsoids indicate the soil location and type. (**B**) Shannon alpha diversity for sugar beet rhizosphere (green) and bulk soil (brown) microbiomes of nine agricultural soils (see Table 2 for soil legend))

The variation in the rhizosphere microbiome composition was compared for a single crop species, sugar beet, grown in all nine agricultural soils. Differences in taxonomic composition between two set samples, sugar beet and the no-crop control (bulk soil), were quantified. An NMDS ordination plot of beta-diversity (based on the Bray-Curtis distance measure) shows that there is variation between the sugar beet rhizosphere (and bulk soil) microbiomes across different soil types (Fig. 5A). The different combinations of soil types and soil locations showed varying diversity distributions. Microbiomes from the same soil types or same location did not clearly cluster together. For example, the silt-clay (SC) soils from two different locations, Hertfordshire and Shropshire (HE & SH) showed distinct communities, as did the two clay (CY) soils from two locations, Yorkshire and Buckinghamshire (YO & BU). However, silt-loam (SL) soils from two different locations, Angus and Shropshire (AN and SH) had overlapping community distributions, and one Bedfordshire (BE) had a separate distribution. Locations HE, YO, BU and BE each group as distinct clusters. This implies that soil type and location combined can act as a driver for distinct microbial communities. Three soil types (SC-HE, CY-BU and SL-BE) give distinctly segregated distributions, indicating that some soil type/location combinations supported unique microbial communities.

A rhizosphere effect, where the presence of the plant type has a strong influence on the microbiota composition, appeared to be more pronounced in some soil type/location combinations than others, e.g. SC-HE compared to CY-BU (Fig. 5A). This was assessed further by calculating alpha diversities (Fig. 5B). Whilst different microbial communities between rhizosphere and bulk soil were evident in some locations only SC-HE showed a significant difference (*p*-value 0.008). (Table S2).

### Case study two: defining a core bacterial rhizosphere microbiome for sugar beet

**Fig. 6:**
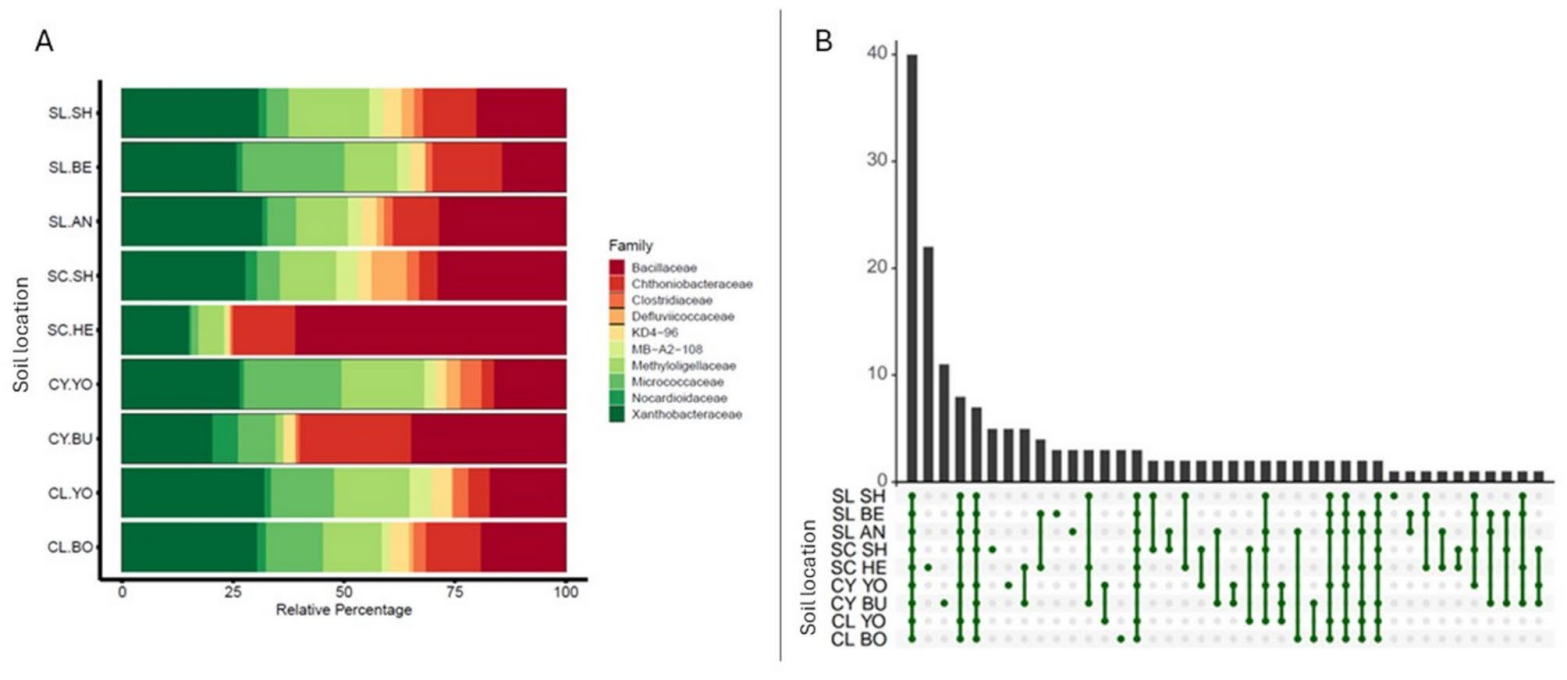
Case study 2: defining a core bacterial microbiome for the sugar beet rhizosphere. (**A**) Stacked bar chart showing relative percentage of ASVs assigned to top 10 taxonomic groups at the family level for the 9 soil types. (**B**) UpSet plot of the intersection of taxa sets across the 9 soil types for the 100 most abundant ASVs assigned at the genus level. The x-axis on the bar chart (upper panel) denotes counts by pattern of genera. One the lower panel each row corresponds to a soil, and each column denotes genus counts. Green dots indicate genera (either common or unique) present in groups. A line edge joins the dots to indicate common genera between soil types. see Table 2 for soil legend)

The concept of a core microbiome refers to a set of microbiota taxa and their functional attributes that are characteristics of one environment. In Case Study One we observed that rhizosphere microbial communities varied between different soil types and locations. Yet, the diversity data also raises the question of what taxa do these communities have in common? Is there a core microbiome characteristic of the sugar beet rhizosphere regardless of the soil type and location? To answer this question, the sugar beet rhizosphere ASVs from the nine agricultural soils were first assigned to bacterial families (Fig. 6A), and the intersections between the 100 most abundant ASVs assigned at the genus level and visualised with an UpSet plot [33] created using UpSetR [34] (Fig. 6B). The UpSet visualisation showed there was variation between the number of sequences according to soil type / location. For example, the rhizosphere microbiome of sugar beet in SC-HE soil contained the highest percentage of *Bacilliaceae* and the rhizosphere microbiome of sugar beet in CY-BU soil had the highest percentage of *Chthoniobacteraceae*.

Forty genera were shown to present in all nine soils from the UpSet plot (Fig. 6B). These 40 genera (Table S3) were defined as the core microbiome for sugar beet rhizospheres at the genus level (for this sample set). Eight of the nine soils had at least one genus that was specific (Fig. 6B, indicated by a green dot with no line edge), only soil CL-YO did not have a unique genus. The only soil type that contained common genera across different locations was clay (CY). These included *Luteitalea*, a member of the *Acidobacteriota* phylum, and *Ohtaekwangia* from the *Bacteroidota* phylum. No common genera were identified in other soil types, clay loam (CL), sandy loam (SL), or silty clay loam (SC).

### Case study three: assessing the impact of soil type and location on the fungal microbiome composition in wheat, with a focus on *Fusarium*

**Fig. 7:**
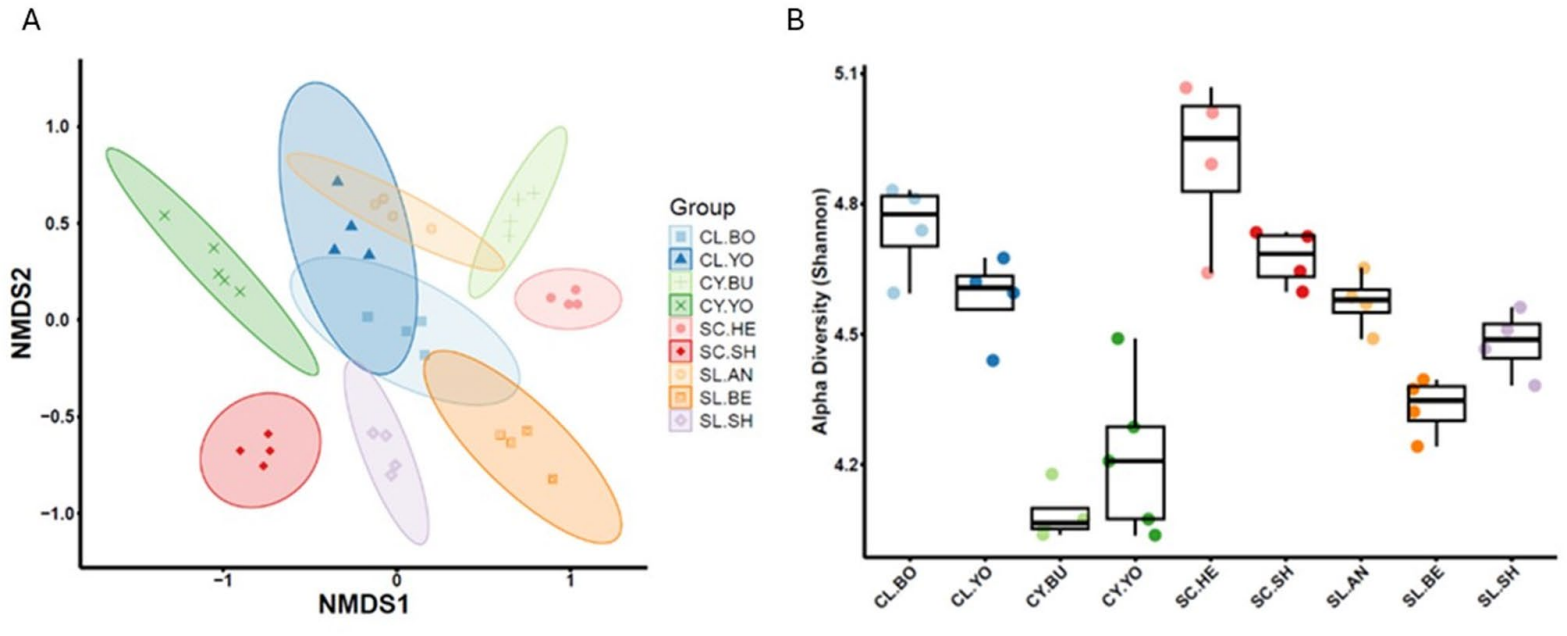
Case study 3: fungal microbiome of spring wheat rhizosphere for nine agricultural soils (**A**) NMDS ordination plot of beta diversity based on the Bray-Curtis distance, (**B**) Shannon alpha diversity (see Table 2 for soil legend))

The fungal rhizosphere microbiome composition was compared for spring wheat across the nine soils. Variation was evident between the rhizosphere microbiomes across all the different soil types as determined from an NMDS ordination plot of beta-diversity (Fig. 7A). There were also significant differences in the variation between the sample sets, as determine by the alpha diversity (Fig. 7B) (Table S4). The clay (CY-BU & CY-YO) soils showed the lowest diversity (Fig. 7B) and distinct community distributions (Fig. 7A). Silty clay loam soil from Hertfordshire (SC-HE) had the highest diversity and a distinct community distribution from silty clay loam from Shropshire (SC-SH). The total number of fungal genera classified for each rhizosphere microbiome ranged between 180 and 259, and a total of 54 common fungi at genus level were identified across all soil rhizospheres. Interestingly, each soil rhizosphere microbiome exhibited unique fungi, ranging from 6 to 37 genera (Table S5).

**Fig. 8:**
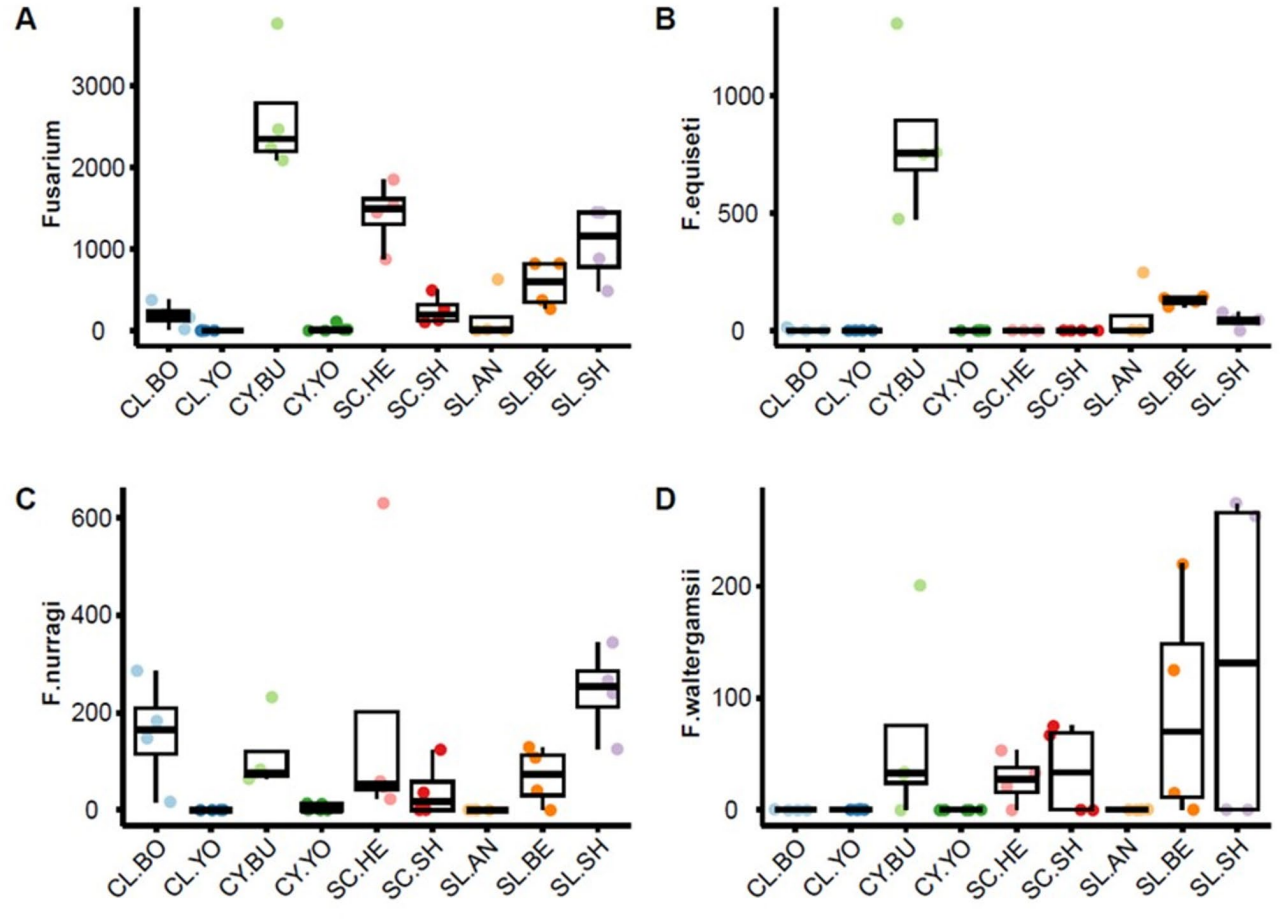
Case study 3: a focus on *Fusarium* genus in wheat rhizosphere: abundance of ASVs classified as *Fusarium* across nine agricultural soils.: (**A**) *Fusarium* genus (all). (**B**) *Fusarium equiseti*. (**C**) *Fusarium nurragi*, (**D**) *Fusarium waltergamsii.* See Table 2 for soil legend

Within the ITS amplicon dataset multiple ASVs were classified to the genus *Fusarium*, corresponding to five species: *F. waltergamsii*, *F. nurragi*, and *F. equiseti* and *F. culmorum*, and *F. tonkinense*. Only one rhizosphere sample included ASVs related to *F. tonkinense* (SC-SH) and one *F. culmorum* (CY-YO), although there were additional ASVs within these samples that could only be classified to the *Fusarium* genus level. Hence, further analysis focused on the ASVs assigned to the *Fusarium* genus and three presumptive *Fusarium* species (Fig. 8) (accurate classification would require validation by qPCR, not carried out in this study). The samples from clay soils in Buckinghamshire (CY-BU) exhibited the highest abundance of total *Fusarium* ASVs, which corresponded to the highest relative level of *Fusarium equiseti* (Fig. 8B). In contrast, the *Fusarium* in silty loam from Shropshire (SL-SH) appeared to comprise more of *F. nurragi* (Fig. 8C) and to some extent *F. waltergamsii* ASVs (Fig. 8D). The abundance of *F. waltergamsii* ASVs was relatively low, with some degree of variation in detection within samples (Fig. 8D).

## Discussion

The UK Crop Microbiome Cryobank is a publicly available resource comprising sequence-based data, meta data and cryo-preserved material related UK crop microbiomes [12]. The sequence-based data (generated from rhizosphere and bulk soil samples for six UK major crops grown in nine agricultural soils), plus associated metadata on the crop varieties and genotypes, soil type, location, heritage, and the soil chemistry, are available from the project’s AgMicrobiomeBase catalogue [31]. The data were obtained and analysed according to suggested standardisation and terminology for microbiome research [35, 36]. Similar sequence-based resources have been described for microbial genomes [37] and eukaryotic sequencing metadata [38], while microbiome analysis tools are accessible in sequence repositories including MGnify [9].

Generation of rhizosphere microbiomes for multiple crops and multiple agricultural soil type / location combinations allows for comparative analyses. Here, specific analyses within three use cases are presented to demonstrate the utility of the data to answer biological questions. The case studies illustrated some of the key influences on the microbiota associated with the UKCMCB resource. The combined effect of soil type and geographical location were shown to be major determinants influencing the structure of microbial communities, mirroring what has been reported elsewhere in crop focused studies (e.g. lettuce, wheat) [39–41].

The focus for the taxonomic composition of the microbiomes was on bacteria as predominant members of the rhizosphere community [42]. The analysis pipeline was developed to account for sequencing and data variability including batch correction and strand merging rates. Rarefaction was not required as there was no large imbalance of sequence reads [43]. Application of the analysis pipeline on both the bacterial (16 S V3-V4 region amplicon) dataset and the partial fungal (ITS-1 region amplicon) dataset revealed that the distribution of the taxonomies varied based on both the host plant and the soil type / location combinations.

While the soil is the main reservoir for the rhizosphere population, root exudation selectively recruits community members, known as the rhizosphere effect [44]. This was evident for the different crops, although to variable extents, affected mostly by the location / soil type combination. The focus on a single crop type, sugar beet, highlighted that enrichment of certain members of the soil bacteria microbiota occurred, especially for one soil type / location (silty clay loam from Hertfordshire) over others, including the clays.

Although this study was not designed to compare locations, an influence of location on the taxonomic diversity was identified by examining the pan- and core-taxonomies, for a single crop (sugar beet). Soil type was also a key driver of diversity, with three out of the four soil types not sharing any of the major taxa. Only the clay soils shared common taxa. This could be due to the soil architecture of clay [45], which has the effect of enriching particular genera compared to other physical structures. Highlighting such diversity within the community composition, even in the presence of a significant rhizosphere effect, raises important questions about function. This is best determined from direct sequencing to identify functional groups coupled to functional phenotype analyses.

Investigation of the fungal composition revealed functional groups of interest. The focus was on ASVs associated with the *Fusarium* species in spring wheat because there are several species within this genus that are phytopathogens, with wheat as a susceptible host [46]. Although ASVs derived from the fungal ITS region are insufficient to define sequence variants to the species level, they allow differential comparisons. There was a strong dependency on soil type / location for the total *Fusarium* population detected as well as on individual ASVs. This could be related to the presence of non-pathogenic species that occur in ‘suppressive’ soils and out-compete other, phytopathogenic species. Notably, bacterial ASVs related to the *Ohtaekwangia* genus were identified as one of the common taxa in the pan-genome assessment, and have previously been identified to potentially suppresses disease-causing *Fusarium* species [47].

Sample metadata, sufficient to allow data re-use is often lacking in datasets deposited in nucleotide repositories, like ENA. This means the re-use of the datasets relies on information held within publications that can be difficult to find and access. To ensure the UKCMCB project data meets FAIR principles (Findable, Accessible, Interoperable, and Reusable) our data catalogue aims to make the link between sample metadata and genomic data easier. The AgMicrobiomeBase catalogue also provides access to our complete bioinformatics workflow code for the amplicon dataset analysis via GitHub. Whilst only the amplicon data has been described here, the complete UKCMCB project has produced multiple additional data types (metagenomic sequence, bacterial isolate sequences and phenotypic data) that will be described elsewhere. As the AgMicrobiomeBase catalogue expands, incorporating further crop and soil datasets, the key to meeting the FAIR principle of reusability will be the sharing of bioinformatics workflows [48] and the development of and adherence to data standards to allow data integration [49]. These aspects are still in development for microbiome research but need to be made a priority: to realise the promise of microbiome data re-use and integration with additional data including functional phenotypes, biochemistry and pathology.

More broadly, the UKCMCB has the potential to allow the analysis of plant growth traits, the generation of synthetic communities (SynComs) and comparisons across multiple microbiome datasets. Additional resources to be included in the UKCMCB are shotgun metagenomic sequences, bacterial isolate phenotype data, culturable bacterial 16 S rRNA and rpoD gene taxonomy, bacterial isolate whole genome sequences and exemplar SynComs. These aspects will be discussed in additional publications.

## Conclusion

In summary, the UKCMCB provides a comprehensive resource relating to crop microbiomes, grown in different UK agricultural soils. The case studies illustrate how the dataset can be queried to answer different biological questions. Since the sequence-based data can be accessed with the extensive soil and crop metadata, they also lend themselves to comparative investigations against other datasets deposited in repositories like MGnify. A meta-analysis on cereal crops found that there was a gap in knowledge and associated datasets for barley in comparison to wheat, rice and maize [5], and our resource helps to fill this gap. The dataset, generated under controlled glasshouse conditions, also serves as a baseline to investigate perturbations, whether from abiotic stress that may arise through climatic changes (drought, heat, salt stress), or biotic stress from infection with pathogens. Equally, since the metadata includes the plant genotype, it has relevance for investigation of alternative races or for breeding strategies, as crop genotypes are known to drive community composition [50]. The metadata within the resource also describes agronomic practices such as the heritage of the soil / location site, in turn allowing comparison of management strategies, whether for application of amendments, pesticides or tillage strategies (e.g. in [51]. Finally, as this dataset focused on the rhizosphere microbiomes, it lends itself to comparison with microbiota derived from other plant tissues, e.g. endophytic compartments or the phylosphere [52].

## Supplementary Information

Below is the link to the electronic supplementary material.

Supplementary Material 1

Supplementary Material 2

## Data Availability

All sequence data has been submitted to the European Nucleotide Archive (ENA) under study accession PRJEB58189 and has been made public. All code used for the analysis of amplicon sequence data is available on GitHub at https://github.com/HuttonICS/agmicrobiomebase. The UKCMCB project data catalogue is publicly available at https://agmicrobiomebase.org/data/.

## Abbreviations

ENA: European Nucleotide Archive
ITS: Internal Transcribed Spacer
rRNA: ribosomal RNA
SynComs: Synthetic communities
UKCMCB: United Kingdom Crop Microbiome Cryobank

## References

[1] Hunter P. Plant microbiomes and sustainable agriculture. EMBO Rep. 2016;17(12):1696–9. 10.15252/embr.201643476 27797857 10.15252/embr.201643476 PMC5283578

[2] Soldan R, Fusi M, Cardinale M, Daffonchio D, Preston GM. The effect of plant domestication on host control of the microbiota. Commun Biology. 2021;4(1):936. 10.1038/s42003-021-02467-6 10.1038/s42003-021-02467-6 PMC8342519 34354230

[3] Ray P, Lakshmanan V, Labbé JL, Craven KD. Microbe to microbiome: a paradigm shift in the application of microorganisms for sustainable agriculture. Front Microbiol. 2020;11. 10.3389/fmicb.2020.622926 10.3389/fmicb.2020.622926 PMC7779556 33408712

[4] Kavamura VN, Mendes R, Bargaz A, Mauchline TH. Defining the wheat microbiome: towards microbiome-facilitated crop production. Comput Struct Biotechnol J. 2021;19:1200–13. 10.1016/j.csbj.2021.01.045 33680361 10.1016/j.csbj.2021.01.045 PMC7902804

[5] Michl K, Berg G, Cernava T. The microbiome of cereal plants: the current state of knowledge and the potential for future applications. Environ Microbiome. 2023;18(1):28. 10.1186/s40793-023-00484-y 37004087 10.1186/s40793-023-00484-y PMC10064690

[6] Castellano-Hinojosa A, Strauss SL, González-López J, Bedmar EJ. Changes in the diversity and predicted functional composition of the bulk and rhizosphere soil bacterial microbiomes of tomato and common bean after inorganic N-fertilization. Rhizosphere. 2021;18:100362. 10.1016/j.rhisph.2021.100362

[7] Newton AC, Hawes C, Hackett CA. Adaptation of winter barley cultivars to inversion and non-inversion tillage for yield and *Rhynchosporium* symptoms. Agronomy. 2021;11(1):30. 10.3390/agronomy11010030

[8] Busby PE, Soman C, Wagner MR, Friesen ML, Kremer J, Bennett A, Morsy M, Eisen JA, Leach JE, Dangl JL. Research priorities for harnessing plant microbiomes in sustainable agriculture. PLoS Biol. 2017;15(3):e2001793. 10.1371/journal.pbio.2001793 28350798 10.1371/journal.pbio.2001793 PMC5370116

[9] Richardson L, Allen B, Baldi G, Beracochea M, Bileschi Maxwell L, Burdett T, Burgin J, Caballero-Pérez J, Cochrane G, Colwell Lucy J, et al. MGnify: the microbiome sequence data analysis resource in 2023. Nucl Acids Res. 2023;51(D1):D753–9. 10.1093/nar/gkac1080 36477304 10.1093/nar/gkac1080 PMC9825492

[10] Matias Rodrigues JF, Schmidt TSB, Tackmann J, von Mering C. MAPseq: highly efficient k-mer search with confidence estimates, for rRNA sequence analysis. Bioinformatics. 2017;33(23):3808–10. 10.1093/bioinformatics/btx517 28961926 10.1093/bioinformatics/btx517 PMC5860325

[11] Thompson LR, Sanders JG, McDonald D, Amir A, Ladau J, Locey KJ, Prill RJ, Tripathi A, Gibbons SM, Ackermann G, et al. A communal catalogue reveals earth’s multiscale microbial diversity. Nature. 2017;551(7681):457–63. 10.1038/nature24621 29088705 10.1038/nature24621 PMC6192678

[12] Ryan MJ, Mauchline TH, Malone JG, Jones S, Thompson CMA, Bonnin JM, Stewart H, Yau PTO, Taketani RG, Clark IM, et al. The UK Crop Microbiome Cryobank: a utility and model for supporting phytobiomes research. CABI Agric Bioscience. 2023;4(1):53. 10.1186/s43170-023-00190-2 10.1186/s43170-023-00190-2 PMC11116202 38800117

[13] Schmidt P-A, Bálint M, Greshake B, Bandow C, Römbke J, Schmitt I. Illumina metabarcoding of a soil fungal community. Soil Biol Biochem. 2013;65:128–32. 10.1016/j.soilbio.2013.05.014

[14] White TJ, Bruns T, Lee S, Taylor J. 38 - Amplification and direct sequencing of fungal ribsomal RNA genes for phylogenetics. In: *PCR Protocols.* Edited by Innis MA, Gelfand DH, Sninsky JJ, White TJ. San Diego: Academic Press; 1990: 315–322.

[15] 16S metagenomic sequencing library preparation. [https://emea.support.illumina.com/downloads/16s_metagenomic_sequencing_library_preparation.html]; 19/09/2023.

[16] The UK Crop Microbiome Cryobank. [https://github.com/HuttonICS/agmicrobiomebase]; 20/03/2025

[17] Andrews S. FastQC: a quality control tool for high throughput sequence data. Available Online at: http://www.bioinformatics.babraham.ac.uk/projects/fastqc; 2010.

[18] FastQC A quality control tool for high throughput sequence data [https://www.bioinformatics.babraham.ac.uk/projects/fastqc/]; 20/03/2025

[19] Ewels P, Magnusson M, Lundin S, Käller M. MultiQC: summarize analysis results for multiple tools and samples in a single report. Bioinformatics. 2016;32(19):3047–8. 10.1093/bioinformatics/btw354 27312411 10.1093/bioinformatics/btw354 PMC5039924

[20] Bolger AM, Lohse M, Usadel B. Trimmomatic: a flexible trimmer for illumina sequence data. Bioinformatics. 2014;30(15):2114–20. 10.1093/bioinformatics/btu170 24695404 10.1093/bioinformatics/btu170 PMC4103590

[21] Bolyen E, Rideout JR, Dillon MR, Bokulich NA, Abnet CC, Al-Ghalith GA, Alexander H, Alm EJ, Arumugam M, Asnicar F, et al. Reproducible, interactive, scalable and extensible microbiome data science using QIIME 2. Nat Biotechnol. 2019;37(8):852–7. 10.1038/s41587-019-0209-9 31341288 10.1038/s41587-019-0209-9 PMC7015180

[22] Martin M. Cutadapt removes adapter sequences from high-throughput sequencing reads. EMBnetjournal. 2011;17(1):10–2. 10.14806/ej.17.1.200

[23] Callahan BJ, McMurdie PJ, Rosen MJ, Han AW, Johnson AJA, Holmes SP. DADA2: high-resolution sample inference from illumina amplicon data. Nat Methods. 2016;13(7):581–3. 10.1038/nmeth.3869 27214047 10.1038/nmeth.3869 PMC4927377

[24] White JR, Roberts M, Yorke JA, Pop M. Figaro: a novel statistical method for vector sequence removal. Bioinformatics. 2008;24(4):462–7. 10.1093/bioinformatics/btm632 18202027 10.1093/bioinformatics/btm632 PMC2725436

[25] Quast C, Pruesse E, Yilmaz P, Gerken J, Schweer T, Yarza P, Peplies J, Glöckner FO. The SILVA ribosomal RNA gene database project: improved data processing and web-based tools. Nucl Acids Res. 2013;41(D1):590–6. 10.1093/nar/gks1219 10.1093/nar/gks1219 PMC3531112 23193283

[26] Nilsson RH, Larsson K-H, Taylor AFS, Bengtsson-Palme J, Jeppesen TS, Schigel D, Kennedy P, Picard K, Glöckner FO, Tedersoo L, et al. The UNITE database for molecular identification of fungi: handling dark taxa and parallel taxonomic classifications. Nucl Acids Res. 2019;47(D1):D259–64. 10.1093/nar/gky1022 30371820 10.1093/nar/gky1022 PMC6324048

[27] Ling W, Lu J, Zhao N, Lulla A, Plantinga AM, Fu W, Zhang A, Liu H, Song H, Li Z, et al. Batch effects removal for microbiome data via conditional quantile regression. Nat Commun. 2022;13(1):5418. 10.1038/s41467-022-33071-9 36109499 10.1038/s41467-022-33071-9 PMC9477887

[28] McMurdie PJ, Holmes S. Phyloseq: an R package for reproducible interactive analysis and graphics of microbiome census data. PLoS ONE. 2013;8(4). 10.1371/journal.pone.0061217 10.1371/journal.pone.0061217 PMC3632530 23630581

[29] Burgin J, Ahamed A, Cummins C, Devraj R, Gueye K, Gupta D, Gupta V, Haseeb M, Ihsan M, Ivanov E, et al. The European nucleotide archive in 2022. Nucl Acids Res. 2022;51(D1):D121–5. 10.1093/nar/gkac1051 10.1093/nar/gkac1051 PMC9825583 36399492

[30] Courtot M, Cherubin L, Faulconbridge A, Vaughan D, Green M, Richardson D, Harrison P, Whetzel PL, Parkinson H, Burdett T. BioSamples database: an updated sample metadata hub. Nucl Acids Res. 2018;47(D1):D1172–8. 10.1093/nar/gky1061 10.1093/nar/gky1061 PMC6323949 30407529

[31] AgMicrobiomeBase. [https://agmicrobiomebase.org/data]; 20/03/2025.

[32] Wilkinson MD, Dumontier M, Aalbersberg IJ, Appleton G, Axton M, Baak A, Blomberg N, Boiten J-W, da Silva Santos LB, Bourne PE, et al. The FAIR guiding principles for scientific data management and stewardship. Sci Data. 2016;3(1):160018. 10.1038/sdata.2016.18 26978244 10.1038/sdata.2016.18 PMC4792175

[33] Lex A, Gehlenborg N, Strobelt H, Vuillemot R, Pfister H. UpSet: visualization of intersecting sets. IEEE Trans Vis Comput Graph. 2014;20(12):1983–92. 10.1109/TVCG.2014.2346248 26356912 10.1109/TVCG.2014.2346248 PMC4720993

[34] Conway JR, Lex A, Gehlenborg N. UpSetR: an R package for the visualization of intersecting sets and their properties. Bioinformatics. 2017;33(18):2938–40. 10.1093/bioinformatics/btx364 28645171 10.1093/bioinformatics/btx364 PMC5870712

[35] Knight R, Vrbanac A, Taylor BC, Aksenov A, Callewaert C, Debelius J, Gonzalez A, Kosciolek T, McCall L-I, McDonald D, et al. Best practices for analysing microbiomes. Nat Rev Micro. 2018;16(7):410–22. 10.1038/s41579-018-0029-9 10.1038/s41579-018-0029-9 29795328

[36] Berg G, Rybakova D, Fischer D, Cernava T, Vergès M-CC, Charles T, Chen X, Cocolin L, Eversole K, Corral GH, et al. Microbiome definition re-visited: old concepts and new challenges. Microbiome. 2020;8(1):103. 10.1186/s40168-020-00875-0 32605663 10.1186/s40168-020-00875-0 PMC7329523

[37] Zhou Z, Alikhan N-F, Mohamed K, Fan Y, Group tAS, Achtman M. The enterobase user’s guide, with case studies on *Salmonella* transmissions, *Yersinia pestis* phylogeny, and *Escherichia* core genomic diversity. Genome Res. 2020;30(1):138–52. 10.1101/gr.251678.119 31809257 10.1101/gr.251678.119 PMC6961584

[38] Challis R, Kumar S, Sotero-Caio C, Brown M, Blaxter M. Genomes on a tree (GoaT): a versatile, scalable search engine for genomic and sequencing project metadata across the eukaryotic tree of life. Wellcome Open Res. 2023;8(24). 10.12688/wellcomeopenres.18658.1 10.12688/wellcomeopenres.18658.1 PMC9971660 36864925

[39] Schlatter DC, Yin C, Hulbert S, Paulitz TC. Core rhizosphere microbiomes of dryland wheat are influenced by location and land use history. Appl Environ Microbiol. 2020;86(5):e02135–02119. 10.1128/AEM.02135-19 31862727 10.1128/AEM.02135-19 PMC7028972

[40] Schreiter S, Ding G-C, Heuer H, Neumann G, Sandmann M, Grosch R, Kropf S, Smalla K. Effect of the soil type on the microbiome in the rhizosphere of field-grown lettuce. Front Microbiol. 2014;5. 10.3389/fmicb.2014.00144 10.3389/fmicb.2014.00144 PMC3986527 24782839

[41] Fernández-Huarte M, Elphinstone JG, Adams IP, Vicente JG, Bhogal A, Watson CA, Dussart F, Stockdale EA, Walshaw J, McGreig S, et al. A DNA-barcode biodiversity standard analysis method (DNA-BSAM) reveals a large variance in the effect of a range of biological, chemical and physical soil management interventions at different sites, but location is one of the most important aspects determining the nature of agricultural soil microbiology. Soil Biol Biochem. 2023;184:109104. 10.1016/j.soilbio.2023.109104

[42] Bulgarelli D, Rott M, Schlaeppi K, van Ver Loren E, Ahmadinejad N, Assenza F, Rauf P, Huettel B, Reinhardt R, Schmelzer E, et al. Revealing structure and assembly cues for *Arabidopsis* root-inhabiting bacterial microbiota. Nature. 2012;488(7409):91–5. 10.1038/nature11336 22859207 10.1038/nature11336

[43] Weiss S, Xu ZZ, Peddada S, Amir A, Bittinger K, Gonzalez A, Lozupone C, Zaneveld JR, Vázquez-Baeza Y, Birmingham A, et al. Normalization and microbial differential abundance strategies depend upon data characteristics. Microbiome. 2017;5(1):27. 10.1186/s40168-017-0237-y 28253908 10.1186/s40168-017-0237-y PMC5335496

[44] Koo BJ, Adriano DC, Bolan NS, Barton CD. Root exudates and microorganisms. In: *Encyclopedia of Soils in the Environment.* Edited by Hillel D. Oxford: Elsevier; 2005: 421–428.

[45] Neal AL, Bacq-Labreuil A, Zhang X, Clark IM, Coleman K, Mooney SJ, Ritz K, Crawford JW. Soil as an extended composite phenotype of the microbial metagenome. Sci Rep. 2020;10(1):10649. 10.1038/s41598-020-67631-0 32606383 10.1038/s41598-020-67631-0 PMC7327058

[46] Walter S, Nicholson P, Doohan FM. Action and reaction of host and pathogen during fusarium head blight disease. New Phytol. 2010;185(1):54–66. 10.1111/j.1469-8137.2009.03041.x 19807873 10.1111/j.1469-8137.2009.03041.x

[47] Ou Y, Penton CR, Geisen S, Shen Z, Sun Y, Lv N, Wang B, Ruan Y, Xiong W, Li R, et al. Deciphering underlying drivers of disease suppressiveness against pathogenic *Fusarium oxysporum*. Front Microbiol. 2019;10. 10.3389/fmicb.2019.02535 10.3389/fmicb.2019.02535 PMC6861331 31781059

[48] Jacobsen A, Kaliyaperumal R, da Silva Santos LOB, Mons B, Schultes E, Roos M, Thompson M. A generic workflow for the data fairification process. Data Intell. 2020;2(1–2):56–65. 10.1162/dint_a_00028

[49] Nijsse B, Schaap PJ, Koehorst JJ. FAIR data station for lightweight metadata management and validation of omics studies. GigaScience. 2023;12. 10.1093/gigascience/giad014 10.1093/gigascience/giad014 PMC9989329 36879493

[50] Bulgarelli D, Garrido-Oter R, Münch PC, Weiman A, Dröge J, Pan Y, McHardy AC, Schulze-Lefert P. Structure and function of the bacterial root microbiota in wild and domesticated barley. Cell Host Microbe. 2015;17(3):392–403. 10.1016/j.chom.2015.01.011 25732064 10.1016/j.chom.2015.01.011 PMC4362959

[51] Luo G, Li L, Friman V-P, Guo J, Guo S, Shen Q, Ling N. Organic amendments increase crop yields by improving microbe-mediated soil functioning of agroecosystems: a meta-analysis. Soil Biol Biochem. 2018;124:105–15. 10.1016/j.soilbio.2018.06.002

[52] Compant S, Cambon MC, Vacher C, Mitter B, Samad A, Sessitsch A. The plant endosphere world – bacterial life within plants. Environ Microbiol. 2021;23(4):1812–29. 10.1111/1462-2920.15240 32955144 10.1111/1462-2920.15240

