# Correction: Introducing the UK Crop Microbiome Cryobank data resource, AgMicrobiomeBase, with case studies and methods on metabarcoding analyses

**DOI:** 10.1186/s40793-025-00846-8

**Published:** 2026-02-10

**Authors:** Payton To Yau, Rodrigo G. Taketani, J. Miguel Bonnin, Helen Stewart, Catriona M. A. Thompson, Ian M. Clark, Tim H. Mauchline, Jacob G. Malone, Matthew J. Ryan, Susan Jones, Nicola Holden

**Affiliations:** 1 Department of Rural Land Use, Scotland’s Rural College, Aberdeen, AB21 9YA UK; 2 Rothamsted Research, West Common, Harpenden, AL5 2JQ UK; 3 CAB International, Silwood Park, Ascot, SL5 7PY UK; 4 John Innes Centre, Norwich Research Park, Norwich, NR4 7UH UK; 5 Information and Computational Science Department, The James Hutton Institute, Dundee, DD2 5DA UK

**Correction: Environmental Microbiome (2025) 20:108** 10.1186/s40793-025-00768-5

Following publication of the original article [1], it came to the author’s attention that the caption of Figure 4 was incorrect. The caption has now been corrected; please be referred to the current version of this caption. The authors thank you for reading this erratum and apologize for any inconvenience caused.
